# Cellular interactions and microenvironment dynamics in skeletal muscle regeneration and disease

**DOI:** 10.3389/fcell.2024.1385399

**Published:** 2024-05-22

**Authors:** Cristina Rodríguez, Filipa Timóteo-Ferreira, Gabriella Minchiotti, Silvia Brunelli, Ombretta Guardiola

**Affiliations:** ^1^ Stem Cell Fate Laboratory, Institute of Genetics and Biophysics “A. Buzzati-Traverso”, CNR, Naples, Italy; ^2^ School of Medicine and Surgery, University of Milano Bicocca, Milan, Italy

**Keywords:** skeletal muscle regeneration, muscle stem cells, macrophages, fibro-adipogenic progenitors, endothelial cells, muscle niche dynamics, muscular dystrophies, regenerative medicine strategies

## Abstract

Skeletal muscle regeneration relies on the intricate interplay of various cell populations within the muscle niche—an environment crucial for regulating the behavior of muscle stem cells (MuSCs) and ensuring postnatal tissue maintenance and regeneration. This review delves into the dynamic interactions among key players of this process, including MuSCs, macrophages (MPs), fibro-adipogenic progenitors (FAPs), endothelial cells (ECs), and pericytes (PCs), each assuming pivotal roles in orchestrating homeostasis and regeneration. Dysfunctions in these interactions can lead not only to pathological conditions but also exacerbate muscular dystrophies. The exploration of cellular and molecular crosstalk among these populations in both physiological and dystrophic conditions provides insights into the multifaceted communication networks governing muscle regeneration. Furthermore, this review discusses emerging strategies to modulate the muscle-regenerating niche, presenting a comprehensive overview of current understanding and innovative approaches.

## 1 Introduction

Skeletal muscle tissue accounts for up to 40% of total body weight (Janssen et al., 2000) and is a complex structure consisting of a highly organized arrangement of muscle fibers embedded within a three-dimensional scaffold composed of collagens, elastins, glycoproteins, proteoglycans, and various other proteins, collectively referred to as the extracellular matrix (ECM) (Csapo et al., 2020). The ECM holds an extensive network of capillaries and nerves, along with various resident cell types, including muscle stem cells (MuSCs), also known as satellite cells, fibroblasts, immune cells and fibro-adipogenic progenitors (FAPs). This complex composition plays a crucial role in maintaining muscle health and supporting motor function (Mukund and Subramaniam, 2020). An exceptional ability of this tissue is the competence for complete regeneration and functional recovery following injury, facilitated by a tightly connected interplay among multiple cellular players, including MuSCs, macrophages (MPs), FAPs, endothelial cells (ECs) and vessel-associated cells, such as pericytes (PCs). The role and functions of MuSCs have been extensively described in recent comprehensive reviews (Schmidt et al., 2019; Relaix et al., 2021; Loreti and Sacco, 2022; Sousa-Victor et al., 2022). Here, we will mainly focus on MPs, FAPs, ECs and PCs, providing definitions for these cell populations, discussing their roles in muscle regeneration in physiological and pathological conditions (muscular dystrophies), and analyzing their reciprocal interaction and crosstalk with MuSCs. Additionally, we will explore current and innovative approaches for recreating and influencing the muscle regenerating niche.

## 2 The skeletal muscle stem cell niche in physiology and disease

The MuSC niche comprises a dynamic and complex microenvironment that includes ECM, growth factors, blood vessels, lymphatic capillaries, nerves, stromal cells, adipose tissue, and various tissue-resident cells (Fuchs and Blau, 2020; Loreti and Sacco, 2022). The myofiber, along with the basal lamina, defines the immediate MuSC niche, providing essential structural support and signaling cues to MuSCs (Hung et al., 2023). In homeostasis, the niche actively supports the quiescent state of MuSCs, which in turn rely on a combination of intrinsic factors and external signals (Goel et al., 2017; Cutler et al., 2022; Hicks and Pyle, 2023; Krauss and Kann, 2023).

Upon injury, disruption in myofiber integrity and the niche architecture alters the structural and biochemical cues that maintain MuSC quiescence. This disruption prompts other cell types, including FAPs, ECs, PCs, and resident and recruited MPs, to gain access to the niche, engaging a reciprocal crosstalk with activated MuSCs and with each other (Wosczyna and Rando, 2018). This complex interplay between cell populations, along with variations in the regenerative microenvironment, significantly influences the transition of MuSCs throughout quiescence, activation, proliferation, self-renewal, and differentiation, ultimately facilitating effective muscle repair (Fuchs and Blau, 2020; Cutler et al., 2022; Krauss and Kann, 2023). Additionally, myofibers release enzymes and myokines such as C-C chemokine ligand 2 (CCL2), Tumor Necrosis Factor-α (TNF-α), and Interleukin-6 (IL-6), influencing muscle regeneration by activating MuSCs in response to damage (Severinsen and Pedersen, 2020; Bivona III et al., 2021).

**FIGURE 1:**
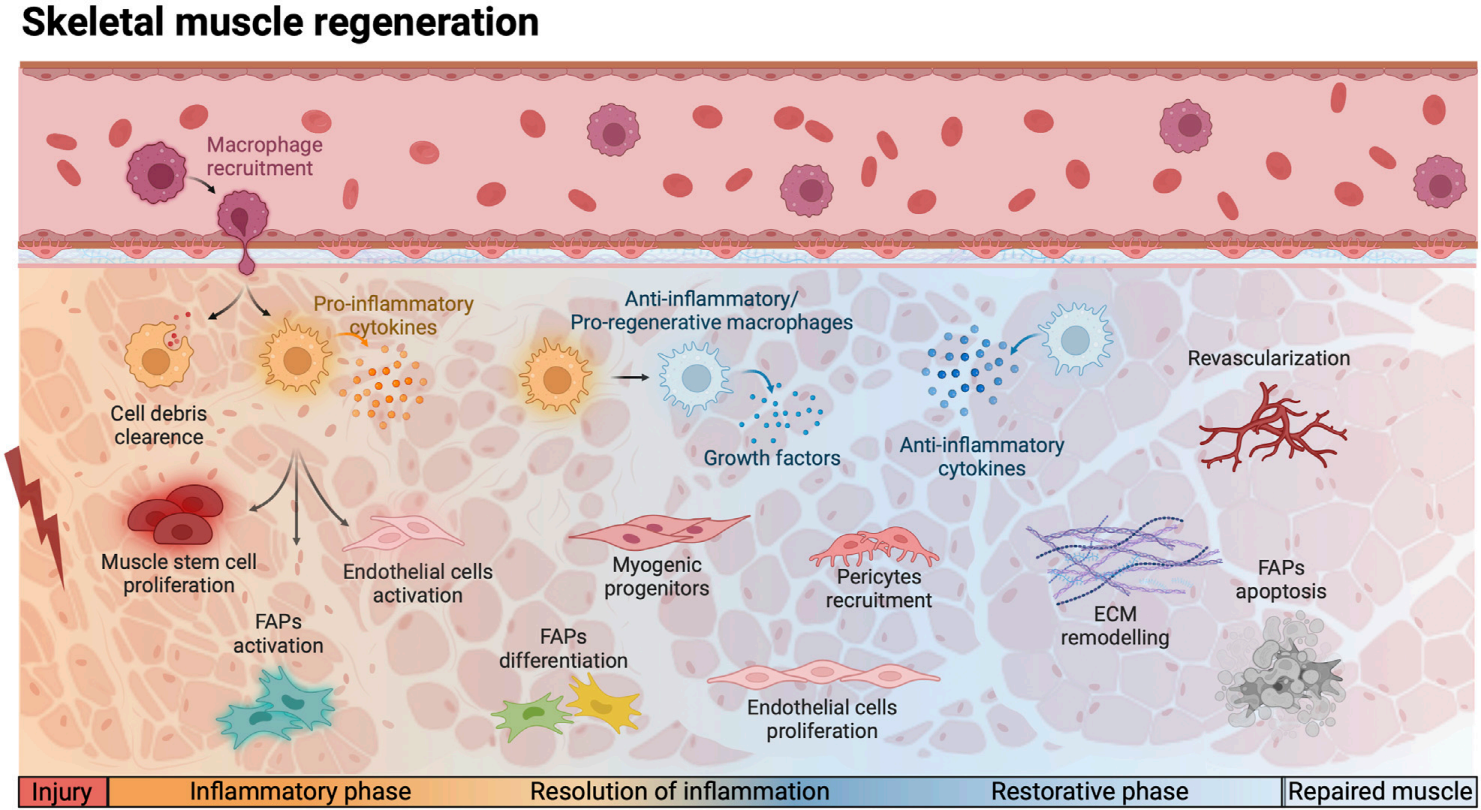
Schematic representation of the cellular and molecular events involved in physiological skeletal muscle regeneration. Following injury, monocytes are recruited to the site of injury, differentiating into pro-inflammatory macrophages and induce a cascade of events. During the inflammatory phase, macrophages are in charge of phagocytosing cell debris and secrete pro-inflammatory cytokines, which induce muscle stem cells proliferation and the activation of endothelial cells and fibro-adipogenic progenitors (FAPs). The resolution of inflammation is triggered by the polarization of macrophages into anti-inflammatory/pro-regeneration phenotype. The secretion of growth factors and anti-inflammatory cytokines promote the differentiation of myogenic progenitors and the proliferation of FAPs and endothelial cells. During the restorative phase, the pericytes are recruited to support endothelial cells and allow the reestablishment of the vascular network, while FAPs undergo apoptosis and the Extracellular Matrix (ECM) is remodeled. This complex interplay ensures efficient muscle repair and functional recovery. Red lightning bolt indicates muscle injury. Created with BioRender.com.

Following muscle injury, a sequential and coordinated number of steps occur. Initially, immune cells, such as circulating monocytes, neutrophils, and leukocytes, are recruited to the damaged site to phagocytose necrotic fibers, dead cells and debris, clearing the area and preparing it for regeneration (Bencze et al., 2012). Subsequently, the secretion of pro-inflammatory cytokines by MPs amplifies the inflammatory response and contributes to promote MuSCs activation (Saclier et al., 2013). Following the initial inflammatory response, there is a transient amplification of the MuSC pool, accompanied by proteolytic modifications and *de novo* deposition of ECM components. These changes are orchestrated through the coordinated actions of MPs, FAPs, ECs, and PCs which secrete the necessary enzymes and proteins (Mashinchian et al., 2018). After the proliferative phase, myogenic progenitor cells fuse to form new muscle fibers. Myogenesis proceeds in parallel to angiogenesis, both stimulated by immune cells that promote tissue repair releasing anti-inflammatory cytokines (Latroche et al., 2017; Mashinchian et al., 2018). Vessel-associated cells, such as PCs and smooth muscle cells, participate in stabilizing the vasculature structure providing essential nutrients and oxygen to newly formed myofibers (Mashinchian et al., 2018; Munroe et al., 2019). Concurrently, the ECM undergoes remodeling to recreate a supportive scaffold for new myofibers, leading to the ultimate repair of the muscle (Schüler et al., 2022) (Figure 1).

In pathological conditions, such as in muscular dystrophies, the disruption of the muscle niche and the lack of coordination among the different cell population compromise the regenerative capacity of the skeletal muscle, and result in chronic inflammation, fat deposition, and fibrosis (Brun et al., 2017; Mashinchian et al., 2018). Muscular dystrophies encompass a group of heterogeneous genetic disorders resulting from mutations in genes encoding different proteins associated with the sarcolemma, ECM, and nuclear membrane (Mercuri and Muntoni, 2013; Smith and Barton, 2018). Duchenne muscular dystrophy (DMD) and Becker muscular dystrophy (BMD), the most common forms of muscular dystrophies, are caused by mutations in the dystrophin gene. DYSTROPHIN is a structural protein and key component of a multiprotein complex that links cytoskeletal proteins to ECM molecules (Ervasti, 2007). Lack of dystrophin results in progressive muscle loss due to prolonged cycles of muscle fiber degeneration and regeneration (Brun et al., 2017).

Emerging evidence indicate that lack of dystrophin directly affects the functionality of MuSCs (Dadgar et al., 2014; Cappellari et al., 2020; Chang et al., 2023). This is revealed by the cell-autonomous defects observed in the asymmetric division, proliferation, migration, and differentiation of dystrophic MuSCs and myoblasts (Dumont et al., 2015; Dumont and Rudnicki, 2016; Chang et al., 2018; Róg et al., 2019; Sun et al., 2020; Gosselin et al., 2022). In addition, studies have shown that the Wnt-TGFβ2 axis upregulates the expression of profibrotic genes, leading to a reduction in the myogenic potential of MuSCs contributing to their dysfunction (Biressi et al., 2014). Indeed, DMD and BMD have been recently classified as secondary satellite cell-opathies, which refer to conditions resulting from genetic mutations affecting both MuSCs and myofiber function (Ganassi et al., 2022). Thus, in muscular dystrophies, alterations in the pathological niche coupled with MuSCs defects, exacerbate disease progression and gradually deteriorate the muscle microenvironment and its functionality (Mashinchian et al., 2018). Over the last few years, there has been an exponential growth in therapeutic strategies to treat DMD and BMD. Efforts have focused not only on the restoration of dystrophin but also on the modulation of muscle cell populations.

## 3 Cellular and molecular crosstalk in muscle regeneration and muscular dystrophies

In this section, we explore the dynamic interplay among various cell populations within the skeletal muscle microenvironment during both the regenerative process and in the context of muscular dystrophies. The complex crosstalk involves an array of different cell populations, including MPs, FAPs, ECs and PCs. We examine their interactions with MuSCs and with each other, shedding light on the multifaceted communication networks that orchestrate acute and chronic muscle regeneration.

### 3.1 Macrophages in physiological muscle regeneration

#### 3.1.1 Skeletal muscle resident macrophages

Skeletal muscle resident MPs play an important role in maintaining tissue homeostasis, contributing significantly to skeletal muscle maintenance, growth, and repair (Uderhardt et al., 2019; Wang et al., 2020). These MPs reside within the interstitial tissue and exhibit a unique transcriptome profile, expressing genes crucial for muscle homeostasis, growth, and regeneration (Wang et al., 2020). Recent studies have shown their involvement in muscle debris clearance following injury (Babaeijandaghi et al., 2022). Additionally, they rapidly respond to tissue microlesions by cloaking them, thereby preventing inflammation and preserving muscle integrity (Uderhardt et al., 2019). Despite progress in understanding their functions, further research is needed to explore their specific roles during muscle regeneration and their interactions within the MuSC niche.

#### 3.1.2 Recruitment and function of infiltrating macrophages

Infiltrating MPs originating from peripheral blood-derived monocytes, are recruited to damaged areas following muscle injury (Arnold et al., 2007). Monocytes exit from the circulation through chemotaxis signaling involving the C-C chemokine receptor type 2 (CCR2) and its main ligand, CCL2 (Tsou et al., 2007). Murine blood monocytes exhibit differential expression levels of CCR2, as well as of lymphocyte antigen six family member C (Ly6C) and C-X3-C Motif Chemokine Receptor 1 (CX3CR1), resulting in the formation of two distinct subsets. Specifically, the Ly6C^high^CCR2^pos^CX3CR1^low^ cell subset that infiltrate the damaged tissue and differentiate into pro-inflammatory MPs (Ly6C^pos^), and Ly6C^low^CCR2^neg^CX3CR1^high^ monocytes, which do not invade the muscle and show patrolling properties (Varga et al., 2013; Chazaud, 2020). Upon arrival to the damaged site, infiltrating MPs play a crucial role in debris and necrotic fiber clearance, as well as in the secretion of inflammatory cytokines. Pivotal studies have investigated the effects of MPs on mouse and human myogenic precursor cells *in vitro* coculture experiments, revealing differential actions depending on the activation/polarization state of MPs that align with those observed in specific MP subsets *in vivo* (Arnold et al., 2007; Saclier et al., 2013). Through the secretion of interleukin-1β (IL-1β), IL-6, TNF-α and vascular endothelial growth factor (VEGF), MPs regulate myogenic precursor cell proliferation and inhibit their premature differentiation (Arnold et al., 2007; Saclier et al., 2013). Moreover, recent findings demonstrated that the MP-secreted cytokine nicotinamide phosphoribosyltransferase (NAMPT) acts through the C-C motif chemokine receptor type 5 (CCR5) receptor expressed by MuSCs to provide a transient stem-cell niche and essential signals for MuSCs proliferation (Ratnayake et al., 2021). MPs also secrete the metalloproteinase-disintegrin ADAMTS1 that suppresses NOTCH1 signaling, an established regulator of MuSCs quiescence, further stimulating MuSCs activation (Du et al., 2017). In addition, the uptake of MP-derived glutamine by MuSCs through the glutamine transporter SLC1A5 enhances MuSCs proliferation and differentiation via mTOR activation (Shang et al., 2020).

#### 3.1.3 Macrophage phenotypic transition and metabolic reprogramming

Several studies have highlighted that muscle regeneration is characterized by sequential steps associated with different MP subsets, illustrating the transition of phenotypically distinct MP populations from pro-inflammatory towards anti-inflammatory/regenerative phenotype (Arnold et al., 2007; Saclier et al., 2013; Varga et al., 2013; Varga et al., 2016a). Traditionally, pro- and anti-inflammatory MPs have been classified as M1 and M2, respectively. However, this conventional classification represents the extremes of a phenotypic *continuum* that cannot be translated to *in vivo* conditions. Indeed, recent advances have shown that during the early stages of acute skeletal muscle regeneration, infiltrating MPs exhibit broad activation, involving simultaneous high expression of both M1 and M2 signature genes. These MPs play a multifaceted role, displaying mixed pro-inflammatory, anti-inflammatory, and pro-regenerative functions to support initial muscle regeneration (Wang et al., 2018). Furthermore, a broader perspective on MP dynamics reveals a specific molecular signature at each step of tissue injury and repair, linked to predictive specialized functions (Varga et al., 2016a). For instance, at early stages of muscle regeneration infiltrating Ly6C + MPs exhibit an inflammatory profile that supports the inflammatory response. Then, these Ly6C + MPs gradually decrease, paving the way for the emergence of Ly6C^−^ MPs presenting anti-inflammatory/regenerative signals. This polarization also induces a shift in the metabolic status of MPs, which transition from a glycolytic metabolism to an oxidative phosphorylation and glutamine metabolism (Varga et al., 2013; Varga et al., 2016a). Several signaling pathways are implicated in the MP phenotypic switch, including insulin-like growth factor 1 (IGF-1) (Tonkin et al., 2015), annexin A1 (ANXA1) through AMP-activated protein kinase (AMPK) (Mounier et al., 2013; McArthur et al., 2020), and the RhoA-ROCK1 pathway regulated by nuclear factor 1 (NFIX) (Saclier et al., 2020). Furthermore, the CCAAT-enhancer-binding proteins (C/EBPs), particularly C/EBPβ, exerts a dual role in skeletal muscle regeneration: as immunomodulator by controlling the expression of pro-inflammatory to anti-inflammatory cytokines in MPs, and by influencing the differentiation, self-renewal, and quiescence of MuSCs (Ruffell et al., 2009; Lala-Tabbert et al., 2021). It has been demonstrated that PAX7+ MuSCs also significantly contribute to the phenotypic transition of MPs during muscle regeneration (Patsalos et al., 2017). At the same time, anti-inflammatory/regenerative MPs stimulate myogenic precursor cell commitment into myocytes and the formation of mature myotubes through the secretion of transforming growth factor-β (TGF-β) and through maintaining low levels of TNF-α (Arnold et al., 2007; Saclier et al., 2013) thus highlighting the complex crosstalk between the 2 cell populations that is essential for normal tissue repair dynamics.

#### 3.1.4 Macrophages and muscle stem cells interactions

It has been reported that muscle MPs express high levels of the fatty acid regulated-transcription factor peroxisome Proliferator-Activated Receptor gamma (PPARγ), whose specific ablation in mouse myeloid lineages leads to impaired muscle repair (Varga et al., 2016b). The underlying mechanism involves the MP-secreted Growth Differentiation Factor 3 (GDF3), a member of the TGF-β family, whose expression is induced in a PPARγ-dependent manner. PPARγ-GDF3 axis regulates myogenic precursor cell differentiation and supports tissue repair through a sensory-regulatory-effector mechanism, possibly alongside other TGF-β family members (Varga et al., 2016b). The crosstalk between MPs and MuSCs during regeneration relies not only on paracrine signals but also on juxtracrine interactions. Direct cell-to-cell contacts occur throughout myogenesis, including tight surface appositions over large areas, long linear distances, and point contacts between MPs pseudopodal extensions and myogenic cell cytoplasmic protrusions (Ceafalan et al., 2018). These contacts appear to be step-specific since pro-inflammatory MPs are observed in close proximity to proliferating MuSCs, whereas anti-inflammatory MPs are preferentially found near differentiating myoblasts (Saclier et al., 2013). Recent advances in tissue-engineered organ-on-chips have contributed to a better understanding of these complex cellular interactions. In a model of engineered skeletal muscle tissue from adult rat myogenic cells, cardiotoxin-induced injury depleted the MuSC pool, leading to progressive tissue degeneration even when treated with pro-regenerative cytokines. However, incorporation of bone marrow-derived MPs into the engineered tissue favored MuSCs proliferation and differentiation, allowing nearly complete muscle repair and sustaining the significance of MPs and MuSCs cell-to-cell interactions during this process (Juhas et al., 2018). Recently, a distinctive subset of MuSCs, identified as immunomyoblasts has been identified by single-cell RNA sequencing and represents a transitional cell state characterized by an enriched expression profile of immune genes (Oprescu et al., 2020). The most significant genes expressed in immunomyoblasts have been also found in activated MuSCs expressing the cell surface protein CRIPTO (TDGF-1, teratocarcinoma-derived growth factor), alongside a comprehensive enrichment of other genes typically associated with immune cells (Guardiola et al., 2023). Furthermore, MuSCs can express a wide range of cytokines and chemokines in response to inflammatory stimuli (Andre et al., 2023). These findings suggest that MuSCs play a more active role than previously thought in influencing immune cell activity.

### 3.2 Macrophages in dystrophic muscle regeneration

#### 3.2.1 Immune dysregulation in dystrophic environment

The onset of dystrophic environments has a detrimental impact on MPs function. Notably, recent research underscores the role of the spleen as a key reservoir of monocytes during chronic inflammation (Swirski et al., 2009). It has been shown that splenic monocytes have a crucial role in modulating inflammation events in dystrophic conditions. Indeed, removal of splenic monocytes reduces early muscle necrosis but disrupts later-stage fiber repair, likely due to delayed MP transition. This suggests that splenic monocytes participate in the initial inflammatory phase but also appear crucial for establishing a pro-regenerative environment (Rizzo et al., 2020). In dystrophic muscles, continuous damage occurs with asynchronous cycles of degeneration and regeneration, disrupting the normal regenerative response (Dadgar et al., 2014). This results in an aberrant accumulation of MPs and chronic inflammation, exhibiting simultaneous upregulation of marker genes typical of specific MP subsets, acting as a trigger for the exacerbation of DMD pathogenesis (Villalta et al., 2009; Mojumdar et al., 2014; Lemos et al., 2015; Juban et al., 2018). Current progress in transcriptomic analysis has allowed a more in-depth understanding of the different MP populations that accumulate in dystrophic muscles. In both mouse models of DMD such as *mdx* mice and D2. *mdx* mice, the most severe *mdx* model, single-cell RNA sequencing analysis revealed an elevated number and more heterogeneous MP populations compared to wild-type mice (Saleh et al., 2022). Notably, none of the MP populations found in the dystrophic muscles corresponds to the traditional definitions of pro- or anti-inflammatory MPs. Instead, the predominant MP signature was characterized by high expression of fibrotic factors (Coulis et al., 2023), thus illustrating MP complexity and dynamic phenotype in muscular dystrophy.

#### 3.2.2 Macrophage dysfunctions

Murine models of DMD exhibit characteristics of trained immunity that worsen the dystrophic condition. This response is triggered by the molecules released from damaged muscle fibers and involves long-term metabolic and epigenetic remodeling in immune cells, primed to overreact. This phenomenon is dependent on Toll-like receptor 4 (TLR4) signaling, a crucial pattern recognition receptor that plays a pivotal role in both innate immunity and the pathophysiology of DMD. Indeed, its activation triggers the production of pro-inflammatory cytokines, exacerbating muscle damage in DMD. TLR4 ablation or inhibition strategies have demonstrated promising results in preclinical models, ameliorating muscle function and reducing inflammation (Giordano et al., 2015; Bhattarai et al., 2022). It has been recently showed that the inhibition of the immunoproteasome, involved in recruiting immune cells to the damaged area, induces a pro-to anti-inflammatory phenotypic switch in the muscle environment, ameliorating the symptoms of aged *mdx* mice (Tripodi et al., 2022).

Recent findings have revealed enhanced expression of canonical markers associated with senescence in both *mdx* and D2. *mdx* mice. This study reveals that dystrophin deficiency leads to the accumulation of senescent and dysfunctional cells, particularly MPs and ECs. Notably, the distribution of these senescent cells varies depending on the mouse model, highlighting potential strain-specific effects (Young et al., 2021). Additional evidence indicates that MPs play a key role in DMD exacerbation as either depleting or hampering the recruitment of MPs reduces muscle fiber degeneration and improves fiber strength in *mdx* mice (Wehling et al., 2001; Mojumdar et al., 2014). However, in a dystrophic mouse model of transient MP depletion, muscle regeneration was compromised, leading to an exacerbation of the dystrophic phenotype, a reduced number of MuSCs, and an impairment of the proliferation/differentiation balance of myogenic progenitors (Madaro et al., 2019). This suggests that the complete depletion of dystrophic MPs could be even more detrimental to muscle repair by interfering with the pro-regenerative crosstalk between the immune system and muscle resident cells (Madaro et al., 2019).

### 3.3 Fibro-adipogenic progenitors in physiological muscle regeneration

#### 3.3.1 Fibro-adipogenic progenitor activation and dynamics

FAPs are mesenchymal progenitor cells located in the interstitial area of skeletal muscle, playing a crucial role in long-term muscle maintenance, regeneration and growth (Wosczyna et al., 2019). In resting conditions, FAPs remain quiescent but, following acute injury, rapidly activate and proliferate, reaching their peak number 72–96 h post-injury. FAP activation results in the transient production of ECM (Joe et al., 2010). The quiescence and activation of FAPs has been associated to the expression of the transcriptional repressor and growth regulator hypermethylated in cancer 1 (Hic1), as its deletion leads to spontaneous mesenchymal progenitor cell expansion (Scott et al., 2019). Further insights into FAP regulation highlighted their orchestration of cellular activation and prevention of excessive over-activation through the production of multiple transcriptional variants of platelet-derived growth factor-alpha (PDGFRα), featuring distinct polyadenylation sites (Mueller et al., 2016). The clearance of proliferating FAPs is essential for a successful return to homeostasis. Indeed, a significant increase in cellular apoptosis and a reduction in the number of FAPs are observed to limit their expansion. In parallel, their differentiation into various lineages is suppressed to prevent the accumulation of fibrosis and/or adipose tissue during the muscle repair process (Lemos et al., 2015). If the FAPs expansion or apoptosis is compromised, they persist in the tissue and differentiate into collagen-producing fibroblasts and adipocytes, contributing to impaired regenerative conditions (Uezumi et al., 2014; Wosczyna et al., 2019). Recent advances in single-cell technologies have revealed FAP heterogeneity, clustering them into different subpopulations with distinct cellular dynamics. Under resting conditions, the main FAP subpopulation expresses the TEK/TIE2 receptor tyrosine kinase (TIE2). However, after injury, a subset of activated FAPs expressing the Vascular Cell Adhesion Molecule 1 (VCAM1**)** emerges. This subset is functionally associated with the inflammatory response (Malecova et al., 2018). However, two other studies have described a different model of FAPs in which PDGFRα positive cells diverge into two trajectories that express either C-X-C Motif Chemokine Ligand 14 (CXCL14) or Dipeptidyl Peptidase 4 (DPP4) in non-injured muscle (Oprescu et al., 2020; Leinroth et al., 2022). Recent studies suggest that FAPs promote muscle regeneration by reactivating a developmental program dependent on the odd-skipped related transcription factor 1 (Osr1). During embryo development Osr1 identifies a population of embryonic FAPs as a subpopulation of interstitial muscle connective tissue (Vallecillo-García et al., 2017). Specifically, Osr1 controls the transcription of ECM genes, such as collagens and components essential for collagenous matrix assembly (Stumm et al., 2018). Adult FAPs express Osr1 at low levels and frequency during homeostasis. Upon injury, Osr1 expression is reactivated (Malecova et al., 2018; Camps et al., 2020; Oprescu et al., 2020; Leinroth et al., 2022), prompting FAPs to enter the cell cycle, undergo apoptosis, and then return to their steady-state pool during regeneration (Stumm et al., 2018). Conditional deletion of Osr1 *in vivo* leads to an impairment in regeneration, resulting in smaller myofibers and altered cytokine and ECM gene expression profiles in FAPs (Kotsaris et al., 2023).

#### 3.3.2 Molecular regulation of fibro-adipogenic progenitor fate specification

The scientific interest in FAP plasticity is progressively increasing, and several molecular mediators underlying their cellular conversion into adipocytes or fibroblasts have been described. For instance, expression of IL-15 in the muscle microenvironment stimulates the proliferation of FAPs through the JAK-STAT pathway. Simultaneously, IL-15 promotes myofiber regeneration by preventing FAP adipogenesis, likely through the induction of desert hedgehog homolog (DHH) signaling (Kang et al., 2018), a repressor of FAPs adipogenesis (Kopinke et al., 2017). Additionally, increased expression levels of adipogenic genes in FAPs is related with an occupancy of the runt-related transcription factor 1 (Runx1) in their regulatory regions. By increasing miR-206 levels, activated FAPs repress Runx1 translation and prevent FAP-to-adipocyte transition thus, limiting fatty infiltration in the muscle (Wosczyna et al., 2021). Another transcription factor that acts as a switch in FAPs transformation is Kruppel-like factor 6 (KLF6). Aside from inducing FAPs autocrine expression of the matrix metalloproteinase 14 (MMP-14), KLF6 is regulated by the expression of miR-22-3p, forming a signaling axis involved in the regulation of FAP differentiation *in vivo* (Lin et al., 2020). Furthermore, *in vitro* cultures of primary murine FAPs have shown that TGF-β1 inhibits FAP adipogenesis while stimulating fibrogenesis. In contrast, bone morphogenetic protein 7 (BMP7), a member of the TGF-β superfamily, promotes FAP adipogenesis but reduces fibrogenesis, suggesting a regulatory function for the TGF-β/BMP signaling pathway (Liu et al., 2022). The enzyme responsible for both TGFβ/BMP gene expression in FAPs and the critical downstream effector is matrix metalloproteinase-13 (MMP-13), as its genetic ablation of blocks TGFβ/BMP signaling in FAP fibro/adipogenesis (Liu et al., 2022). Recent findings revealed that different levels of Stem Cell Antigen-1 (SCA-1) influence FAPs fate. Specifically, while SCA^Low^ FAPs are more prone to a fibrogenic fate, SCA1^High^ FAPs tend to be adipogenic by expressing higher levels of PPARγ (Giuliani et al., 2021). Indeed, PPARγ is a key regulator of adipogenesis and MuSCs function during muscle regeneration. In PPARγ knock-out (KO) mice, FAPs completely lose the ability to differentiate into adipocytes after injury and this correlates with the absence of intramuscular lipid accumulation (Dammone et al., 2018). The loss of PPARγ also impairs the proliferation and myogenic commitment of MuSCs during muscle regeneration. However, this is likely an indirect effect since isolated PPARγ KO MuSCs do not show significant defects in proliferation or differentiation *in vitro* (Dammone et al., 2018). Depletion of FAPs in the PDGFRα knock-in mouse model has revealed the essential role of these cells in muscle regeneration and homeostasis. This depletion resulted in muscle atrophy, weakness, and a reduction in MuSCs, highlighting the critical function of FAPs in maintaining muscle health (Wosczyna et al., 2019; Uezumi et al., 2021).

#### 3.3.3 Interplay between fibro-adipogenic progenitors and macrophages in tissue remodeling

Despite the intrinsic mechanisms underlying FAP fate specification, a reciprocal and functional interplay with MuSCs has proven to be equally essential. Indeed, FAPs located near injured myofibers support MuSC proliferation and create a transient pro-differentiation niche for myogenic progenitors (Joe et al., 2010; Fiore et al., 2016). This feature appears to be characteristic of a small subset of FAPs that express the zinc finger protein GLI1, a crucial mediator of Hedgehog signaling. GLI1^+^ FAPs expand after muscle injury to ensure efficient regeneration by regulating myofiber size and preventing fat deposition (Yao et al., 2021). In turn, muscle fibers directly repress FAP adipogenesis (Uezumi et al., 2010). In addition to the important role of NOTCH in the modulation of MuSC activation and differentiation, it was recently demonstrated that it can also suppress the adipogenic capacity of FAPs (Marinkovic et al., 2019). Interestingly, beyond the capacity to differentiate into fibrogenic and adipogenic cells, FAPs may also play a role in heterotopic ossification in the muscle by differentiating into osteogenic or chondrogenic cells (Eisner et al., 2020). The interplay between FAPs and MPs in tissue remodeling has received relatively limited attention until recently. Yet, FAPs have been identified as the main cellular target of TGF-β1 secreted by MPs following injury (Stepien et al., 2020; Nawaz et al., 2022). The existence of a dynamic communication between FAPs and MPs, which is mediated by TGF-β1, is further supported by the observation that the regenerative response improves when either TGF-β1 is specifically deleted in myeloid cells or when CD206^+^ anti-inflammatory MPs are depleted. This can be attributed to the reduced proliferation and differentiation of FAPs that are responsible for ECM deposition and fibrosis (Stepien et al., 2020; Nawaz et al., 2022). Interestingly, FAPs in turn regulate, in part, MP polarization through the expression of Osr1. Indeed, conditional depletion of Osr1 gene in FAPs substantially increases the number of pro-inflammatory MPs while reducing anti-inflammatory MPs (Kotsaris et al., 2023). This multifaceted functionality positions FAPs as master regulators of MuSCs function and crucial contributors to the orchestration of muscle regeneration.

### 3.4 Fibro-adipogenic progenitors in dystrophic muscle regeneration

#### 3.4.1 Fibro-adipogenic progenitor dysfunction in dystrophic muscle regeneration

In a dystrophic muscle environment, the aberrant persistence of FAPs prevents tissue clearance and contributes to fibrosis, fat deposition, and impaired muscle regeneration (Uezumi et al., 2014). In spite of the main consequences of dystrophin deficiency in DMD being fiber degeneration and chronic inflammation, aberrant replacement of muscle by ECM significantly contributes to disease progression. Indeed, increased levels of TGF-β in DMD muscles have been found to contribute to aberrant ECM deposition due to increased FAP proliferation (Contreras et al., 2019). The TGF-β released into the stromal space downregulates PDGFRα expression in FAPs, inhibiting their adipogenicity and promoting myofibroblast differentiation (Contreras et al., 2019). A single-cell RNA sequencing study revealed an altered stromal cell composition in both dystrophic and severely dystrophic skeletal muscles, characterized by a reduced abundance of cell subtypes compared to healthy muscles. Notably, the study showed an increased prevalence of the activated FAP subtype expressing the chemokine C-X-C motif ligand 5 (CXCL5) in mdx and D2. *mdx* mice compared with wild-type mice (Saleh et al., 2022). In dystrophic environments, FAPs also display mitochondrial dysfunction with increased glycolysis (Reggio et al., 2020), altered differentiation potentials *in vivo* and *ex vivo* compared to their wild-type counterparts (Mozzetta et al., 2013), and insensitivity to NOTCH-mediated inhibition of adipogenic differentiation (Marinkovic et al., 2019). Additionally, miRNA expression profile in FAPs from DMD patients differ from that of healthy muscle. In this context, miR-214-3p has been identified as a key player in the regulation of TGF-β-induced FAP activation by targeting the fibroblast growth factor receptors (FGFR) signaling pathway (Arrighi et al., 2021). Genetic and pharmacological studies have highlighted FAPs as potential therapeutic targets in DMD. Depletion of FAPs proliferative fraction (PDGFRβ^+^) reduces muscle damage and endurance loss due to decreased infiltration of pro-inflammatory MPs and reduced expression of *Tgfβ1* (Gao et al., 2021). Furthermore, it has been recently reported that targeting the TGF-β signaling in D2. *mdx* mice reduce muscle degeneration, calcification and fibrosis by blocking FAP accumulation (Mázala et al., 2023). Similarly, inhibition of Rho-kinase, which is highly expressed in dystrophic muscles (Zhao et al., 2003) reduces FAP PDGF-AA expression, improves muscle function, and reduces fibrosis (Fernández-Simón et al., 2022). Aging in dystrophic environments also affects FAP population, reducing their total number and increasing the number of PPARγ-expressing cells, indicating an increased adipogenic commitment of FAPs in this condition. This is regulated by CD45^+^ cells in *mdx* mice; indeed, young but not old CD45^+^ cells inhibit the adipogenic differentiation pathway, indicating that the young *mdx* microenvironment is more efficient in inhibiting FAPs adipogenesis (Giuliani et al., 2021). It is worth noting that FAPs exhibit muscle-type specificity and distinct responses in muscular dystrophy. The diaphragm and quadriceps of *mdx* mice display different transcriptomes, with FAPs in the diaphragm showing higher levels of gene and protein expression compared to those in the quadriceps. Notably, the diaphragm also exhibits a higher percentage of proliferating and apoptotic FAPs, along with a downregulation of TNF-α signaling (Wang et al., 2023).

#### 3.4.2 Interplay between fibro-adipogenic progenitors and other muscle cell populations

The expansion of FAPs post-injury is linked to their crosstalk with other muscle cell populations, playing a pivotal role in orchestrating proper muscle healing processes. For example, soluble factors from myogenic progenitors activate the phosphatidylinositol 3-kinase PI3K/AKT pathway in FAPs stimulating proliferation, while myotubes regulate FAP differentiation through pro-fibrogenic and anti-adipogenic factors. This interaction is disrupted in aged and DMD patients, highlighting a crucial interplay in adipocyte and myofibroblast accumulation in dystrophic and aging muscle contexts (Moratal et al., 2019). Clearance of FAPs is an important event for a correct muscle healing and is, in part, regulated by the switch from TNF-producing inflammatory MPs to pro-regenerative MPs expressing TGF-β1 in the muscle regenerating niche. While TNF is responsible for efficient FAP clearance, the subsequent wave of TGF-β1 blocks the pro-apoptotic effects of TNF and ensures an efficient tissue regeneration (Lemos et al., 2015). Indeed, in dystrophic muscle environments, where a large subset of MPs expresses both TNF and TGF-β1, this ordered transition is disrupted and the muscle presents higher number of FAPs that is correlated with increased fibrosis (Lemos et al., 2015), which also results in a poor regenerative myogenesis and greater muscle degeneration (Mázala et al., 2023). Additionally, in dystrophic muscles dysregulated MPs secrete higher levels of growth factors and latent TGF-β1 that is subsequently activated by FAP-secreted enzymes, further promoting FAPs proliferation and ECM component release (Juban et al., 2018; Wang et al., 2023). Furthermore, *mdx* mice present a chronic activation of the pro-fibrotic factor osteopontin, which has been suggested to mediate, at least in part, the communication between FAPs and MPs. Elevated levels of osteopontin and collagen have also been identified in human DMD biopsies, providing additional support for the role of MP-FAP fibrogenic axis in DMD pathogenesis exacerbation (Coulis et al., 2023).

### 3.5 Endothelial cells and pericytes in physiological muscle regeneration

Skeletal muscle contains the highest microvascular mass capable of adapting to environmental and physiological variations (Latroche et al., 2015a). Within the muscle tissue, endomysial capillaries formed by ECs are arranged in parallel to the myofibers, with usually three to four adjacent myofibers disposed close to 5-10 capillaries (Lo et al., 2003). In this complex system, ECs play a key role in regulating local vascular tone and maintaining homeostasis in vascular biology. After muscle damage, these cells are promptly activated to vascularize the regenerating tissue, provide nutrients and oxygen through angiogenesis, and increase vessel permeabilization, allowing the recruitment of immune cells (McLennan, 1996).

#### 3.5.1 Endothelial and muscle stem cell coordination

The coordination between angiogenesis and myogenesis is indispensable for the full restoration of skeletal muscle after injury. ECs establish close contact with quiescent, proliferating, and differentiating MuSCs (Christov et al., 2007), and interact through the release of several factors. *In vitro* co-culture experiments, in the absence of cell-to-cell contacts, have shown that ECs promote myogenic cell growth by secreting soluble factors, including IGF-1, hepatocyte growth factor (HGF), basic Fibroblast Growth Factor (bFGF), PDGF-BB, and VEGF (Christov et al., 2007). Furthermore, ECs secrete apelin, oncostatin M, and periostin, promoting myogenic precursor cell migration, proliferation, and differentiation and orchestrate the coupling between myogenesis/angiogenesis during repair (Latroche et al., 2017). Recently, the transcriptional enhanced associate domain 1 (Tead1)-Apelin axis has been implicated as a novel regulator of endothelial remodeling via paracrine communication with myofibers (Lee et al., 2022). Single-cell RNA sequencing of regenerating muscles and *in vitro* cultures of ECs with myotubes revealed that the Apelin receptor is enriched in ECs, whereas myogenic cells mainly express Tead1. Building on this knowledge, further *in vivo* and *in vitro* experiments proved the existence of a Tead1-Apelin axis, in which Tead1 regulates apelin secretion by myogenic cells, thereby controlling ECs remodeling (Lee et al., 2022). Moreover, the angiogenic growth factor angiopoietin-1 (ANG-1), which is secreted in the post-injury muscle environment, also contributes to the maintenance of the endothelium and supports myogenesis by enhancing myoblasts proliferation and differentiation (Mofarrahi et al., 2015). The extent of myogenic cell differentiation has also been directly correlated with capillary elongation and lumenization, which further highlights the tight coordination between both processes (Latroche et al., 2017).

MuSCs and differentiating myogenic cells reciprocally stimulate angiogenesis and sustain regeneration by secreting VEGF (Christov et al., 2007; Bryan et al., 2008) through β-catenin (Kim et al., 2006) and the hypoxia-inducible factor 1 alpha (HIF-1α) pathway (Rhoads et al., 2009). This suggests a feed-forward mechanism where VEGF is involved in orchestrating both angiogenesis and myogenesis. Moreover, myocyte-derived VEGF is crucial for the formation of new capillaries after muscle overload via angiotensin II signaling (Gorman et al., 2014). CXCL12 is released by myofibers upon stretching, promoting angiogenesis and revealing a crosstalk between myofibers and ECs. However, it has been demonstrated that deletion of CXCL12 does not significantly affect angiogenesis in the muscle (Yamada et al., 2019), thus indicating the involvement of other factors in regulating this process. Both *in vitro* and *in vivo* studies have revealed an important function of ECs in regulating MuSCs quiescence and self-renewal. This is mediated by the secretion of VEGF-A by MuSCs, which recruits ECs to create a vascular niche. In turn, this niche expresses the delta like canonical Notch ligand 4 (Dll4) that activates NOTCH signaling pathway in MuSCs and promotes their quiescence (Verma et al., 2018). Complementary to these findings, single-cell RNA sequencing analysis revealed that Dll4 is exclusively expressed in ECs in the muscle (Fujimaki et al., 2022). Moreover, the EC-derived soluble form of Dll4 activates Notch2 receptors on the myofibers without direct cell–cell contact (Fujimaki et al., 2022). Moreover, when ECs become dysfunctional, the secreted factors negatively affect MuSC expansion, differentiation, and fusion into myotubes (Kargl et al., 2019), further confirming the important role of ECs in influencing myogenesis.

#### 3.5.2 Multiple functions of pericytes in muscle repair

PCs are a group of resident mesenchymal cells within the microvasculature and often overlooked. These endothelial-associated cells are frequently confused with vascular smooth muscle cells because of their common location and involvement in the constriction and dilation of blood vessels, which is achieved through the secretion of ANG-1 and binding to the angiopoietin one receptor Tie-2 in ECs (Wakui et al., 2006). However, it has been demonstrated that PCs represent a second myogenic precursor phenotypically different from MuSCs (Dellavalle et al., 2007). Lineage tracing studies performed during pre- and post-natal development established that skeletal myogenesis is an intrinsic fate of PCs (Dellavalle et al., 2011). They contribute to the smooth muscle layer of blood vessels, generate MuSCs in the early post-natal period and muscle fiber development in both cardiotoxin-induced injury and chronic muscular dystrophy (Dellavalle et al., 2011). In addition, PCs create a niche that regulates MuSCs quiescence through ANG-1 and stimulate muscle growth through IGF-1 (Kostallari et al., 2015). Therefore, MuSCs are not the only muscle resident cells directly responsible for myogenesis, although what regulates PCs towards a myogenic fate is still far to be completely understood. Dysregulation of growth factors, for example, can negatively impact pericytes. VEGF can trigger both normal and aberrant angiogenesis in the muscle, in a dose-dependent manner, with elevated levels being associated with the depletion of vascular PCs (Gianni-Barrera et al., 2013). However, co-expression of PDGF-BB and VEGF modulates the VEGF receptor 2 (VEGF-R2) signaling, restraining ECs proliferation and preventing the occurrence of abnormal angiogenesis, even at high concentrations of VEGF. Furthermore, this correlates with PC retention on endothelial structures during the initial stages of VEGF-induced vascular enlargement, which suggests that PDGF-BB plays a vital role in maintaining vascular stability (Gianni-Barrera et al., 2018). Recently, the mechanosensitive ion channel protein Piezo1 has been reported to be critical in the collaborative relationship between muscle ECs and PCs, and in maintaining muscle capillarity (Bartoli et al., 2022). Indeed, EC conditional depletion of Piezo1 induces microvascular EC apoptosis and PC regression (Bartoli et al., 2022). Although ECs and PCs have always been considered two different and independent populations, a recent single-cell RNA sequencing study described a new muscle population that co-expresses PCs and ECs markers, termed EC- like PCs (ELPCs) (Cameron et al., 2022). RNA velocity analysis also revealed that ELPCs appeared as an intermediate state, consistent with a transition state between ECs and PCs, which was supported by latent time analysis. The presence of ELPCs was also confirmed in skeletal muscle biopsies (Cameron et al., 2022).

### 3.6 Endothelial cells and pericytes in dystrophic muscle regeneration

#### 3.6.1 Impaired angiogenesis and endothelial cells dysfunction

Impaired angiogenesis in dystrophic animals has been extensively demonstrated (Loufrani et al., 2004; Matsakas et al., 2013; Palladino et al., 2013; Podkalicka et al., 2021) with dysfunction of blood vessels affecting not only the oxygen and nutrient supply but also the mechanical function of the muscle fibers (Latroche et al., 2015b). It is noteworthy that dystrophin is also expressed in ECs, implying that the function of these cells could be compromised in patients with DMD and potentially contribute to the progression of the disease (Loufrani et al., 2001). In fact, in a canine DMD model it has been demonstrated that dystrophin plays a crucial role in maintaining the structure and function of vascular endothelium and smooth muscle, with vascular defects contributing to disease pathogenesis (Kodippili et al., 2021). In *mdx* mice, ECs exhibit reduced migration, proliferation, and tube formation *in vitro,* compared to ECs from wild-type mice. The dystrophic ECs also display increased apoptosis and higher activity of senescence-associated β-galactosidase (Palladino et al., 2013) which correlates with the observation that the initial cell types that undergo senescence in DMD are ECs and MPs (Young et al., 2021). When co-cultured with primary myoblasts, ECs from *mdx* mice are also less effective at supporting myoblast proliferation (Palladino et al., 2013). Recently, single-nuclei RNA sequencing data from skeletal muscles of *mdx* and D2. *mdx* mice revealed that ECs are enriched in pathways related to fibrillar collagen production, while pathways related to signaling receptor activity and molecular transduction were suppressed (Saleh et al., 2022). Conversely, crucial genes responsible for EC function were downregulated, indicating potential functional and signaling deficits together with increased propensity to fibrosis (Shen et al., 2023). At the same time, single-cell RNA sequencing showed that the proportion of distinct capillary EC populations changes drastically between healthy and dystrophic muscles, presenting a reduced number in *mdx* and D2. *mdx* mice, with gene expression analysis showing an upregulation of ECM-related genes, as well as genes associated with platelet activation and aggregation in severely dystrophic ECs (Saleh et al., 2022). Furthermore, it has been reported that the PC population is reduced in *mdx* mice, suggesting that this may contribute to increased vascular permeability and other vascular abnormalities that are observed in DMD (Podkalicka et al., 2021). Researchers have explored the possibility of modulating angiogenesis as a therapeutic strategy for DMD. VEGF has been considered as a potential treatment for DMD, although several drawbacks have been identified, including limited half-life of the protein and side effects like the risk of vascular leakage, disorganized angiogenesis, and increase in fibrotic markers (Gutpell and Hoffman, 2015). An alternative approach has involved targeting the VEGF receptor Flt-1 (VEGF-R1) that acts as a decoy for VEGF (Verma et al., 2019). In contrast, when Flt-1 was specifically deleted in post-natal ECs, it increased vascular density, MuSCs number, and improved DMD-associated muscle pathology without significant adverse effects (Verma et al., 2019). Furthermore, both anti-FLT-1 peptides and monoclonal antibodies promoted vascularization, blood flow, reduced fibrosis, and increased muscle strength in *mdx* mice (Verma et al., 2019; Bosco et al., 2021). These recent findings are in line with earlier studies showing that Flt-1 haploinsufficiency in *mdx* mice increases capillary density and reduces fibrosis, calcification, and membrane permeability while increasing muscle force function (Verma et al., 2010). Taken together, these results suggest that targeting FLT-1 to improve angiogenesis in dystrophic muscles could be a promising therapeutic strategy without the adverse effects encountered with VEGF treatment. Interestingly, the injection of human adipose tissue-derived PCs improves the life span of dystrophin/utrophin double KO mice (Valadares et al., 2014). In addition, PC-like cells, known as mesangioblasts, have been reported to improve muscle regeneration in various types of muscular dystrophy (Cossu et al., 2015).

### 3.7 Endothelial cell crosstalk with macrophages and fibro-adipogenic progenitors in muscle regeneration

#### 3.7.1 Endothelial cell crosstalk with macrophages

The cellular crosstalk established by ECs and PCs with other muscle populations is crucial for orchestrating a successful repair process. Monocyte chemoattractant protein-1 (MCP-1) and its receptor CCR2 that are involved in monocyte/MPs recruitment to the sites of injury, are essential for ECs activation and support of angiogenesis (Rowe et al., 2014). In the absence of CCR2, the regenerating muscle presents lower levels of tissue VEGF, leading to reduced capillary formation and impaired muscle healing (Ochoa et al., 2007). Furthermore, Wnt signaling in MPs acts in an autocrine manner to stimulate VEGF production, therefore enhancing endothelial permeability (Tusavitz et al., 2020). On the other hand, restorative MPs secrete oncostatin M that stimulates the coupling between myogenesis/angiogenesis (Latroche et al., 2017). A recent study highlights the close interconnection between these two cellular populations, revealing a novel mechanism of metabolic angiocrine signaling that influences MP phenotypic switch, ultimately contributing to tissue homeostasis and regeneration. The authors demonstrated that the specific loss of the glycolytic enzyme 6-phosphofructo-2-kinase/fructose-2, 6-biphosphatase 3 (*pfkfb3*) in ECs reduces EC lactate secretion, hindering ischemic hindlimb revascularization and muscle regeneration by impairing the acquisition of a proangiogenic phenotype by MPs. Furthermore, the study shows that EC-derived lactate acts as a signaling molecule to enhance VEGF secretion, thereby guiding MPs toward an M2-like phenotype through monocarboxylate transporter 1 (MCT1), promoting muscle regeneration (Zhang et al., 2020).

#### 3.7.2 Endothelial-to-mesenchymal transition (EndMT) and muscle repair

A significant reduction in the number of MPs or a functional impairment may lead to failed muscle regeneration (Summan et al., 2006; Segawa et al., 2008; Liu et al., 2017). In fact, depletion of infiltrating MPs following skeletal muscle injury promotes the differentiation of endothelial-derived progenitors into mesenchymal-like cells, contributing to an overall increase in collagen deposition and abnormal tissue remodeling (Zordan et al., 2014). This trans-differentiation of ECs to mesenchymal-like cells is known as endothelial-to-mesenchymal transition (EndMT), a complex biological mechanism in which ECs progressively lose their endothelial phenotype and cobble-stone like shape to acquire the expression of mesenchymal-related genes and a more elongated/spindle appearance. Although EndMT was originally recognized in the context of embryonic development, it has also been observed to occur postnatally in specific circumstances, such as angiogenesis, where partial EndMT has been reported (Welch-Reardon et al., 2014). However, uncontrolled EndMT has been linked to various fibrotic diseases (Zeisberg et al., 2007) and compromised muscle healing (Zordan et al., 2014; Iavarone et al., 2020). Interestingly, a mouse parabiosis model has revealed that the ability to undergo EndMT is not restricted to local ECs. Circulatory ECs recruited to the site of injury can also undergo this process and contribute to the diseased muscle environment (Agarwal et al., 2016). The specific contribution of the pro- and anti-inflammatory MPs to EndMT is still debated. Interestingly, recent studies have shown that myeloid lineage-specific CRIPTO knock-out mice exhibit reduced accumulation of anti-inflammatory CD206^+^ MPs in acute muscle injury and chronic disease, which correlates with increased EndMT and fibrosis. These findings shed light on the distinct role played by various MP subtypes in regulating EndMT, thereby contributing to restrain the aberrant accumulation of ECM (Iavarone et al., 2020). Recent research provides compelling evidence that in dystrophic environments loss of EC biochemical and phenotypic identity, through mesenchymal transformations, causes severe deficits in myogenesis and angiogenesis, and exacerbates regenerative impairments (Pessina et al., 2015). Both murine and human DMD muscles present cells co-expressing mesenchymal and endothelial markers, along with increased activation of the P-SMAD2/3 signaling (TGF-β associated pathway). Consistently, as fibrosis and disease severity progress with age, a simultaneous increase in active TGF-β levels is observed, possibly produced by inflammatory cells and FAPs (Pessina et al., 2015). Recent single-cell RNA sequencing analysis have further supported these findings identifying a subset of capillary ECs exhibiting upregulated expression of collagen-related genes (*Col1a1*, *Col3a1*, *Col4a1*, *Col6a1*) and fibronectin in DMD muscles (Saleh et al., 2022). Assessment of possible interactions responsible for the observed gene expression changes in ECs within dystrophic environments, identified that the main ligands driving ECM expression in D2. *mdx* ECs were TGF-β, ostepontin, TNFα and IL-1β. These ligands interactions were attributed to stromal cells and MPs within the dystrophic muscle microenvironment and TGF-β pathway was identified as the main upstream signal driving EC dysregulation (Saleh et al., 2022). This suggests that in dystrophic environments, a portion of fibrogenic cells may arise from EndMT due to the TGF-β-enriched environment, contributing to increased ECM deposition.

**FIGURE 2:**
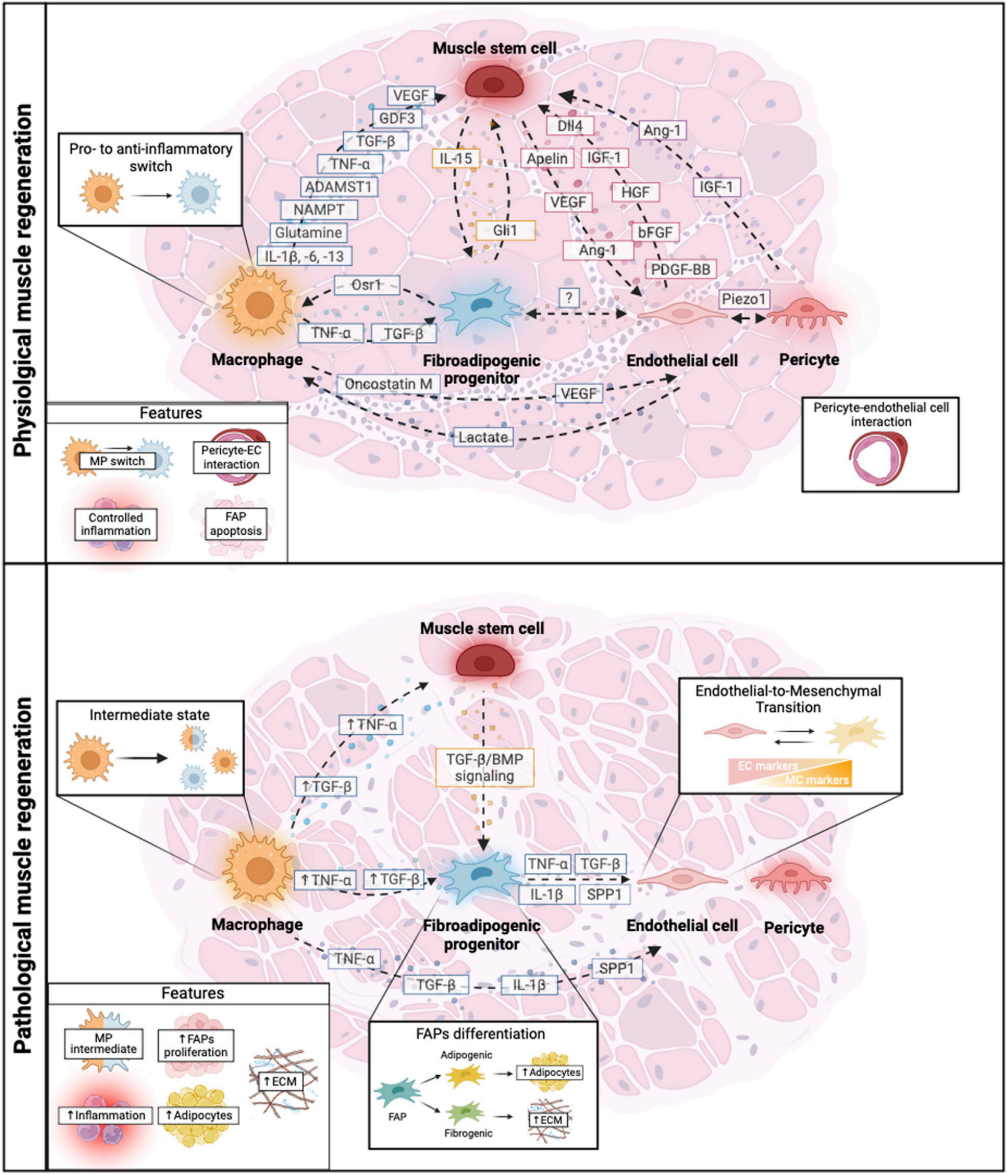
Schematic representation of the crosstalk among key cellular players during physiological and pathological muscle regeneration. Muscle regeneration is mediated by the crosstalk between muscle cell populations such as muscle stem cells, macrophages (MPs), fibro-adipogenic progenitors (FAPs), endothelial cells, pericytes and their reciprocal cross talk. In physiological conditions, there is a tight balance of growth factors secreted by different muscle populations that orchestrate muscle regeneration allowing different processes such as controlled inflammation, MP polarization (MP switch), correct pericyte-endothelial cell interaction and FAPs function. However, during pathological muscle regeneration, this balance is disrupted leading to an altered crosstalk of the muscle cell populations. This leads to increased inflammation presenting MP intermediate states, uncontrolled Endothelial-to-Mesenchymal Transition, FAPs proliferation and differentiation into adipogenic or fibrogenic cells, resulting in altered ECM deposition and ultimately influencing disease exacerbation. Dashed arrows represent cell-to-cell communication. Solid arrows represent upregulated molecular factors in pathological conditions. Created with BioRender.com.

The reviewed crosstalk between the different muscle populations (MuSCs, MPs, FAPs, ECs, and PCs) during muscle regeneration in health and disease including the main growth factors and signaling pathways involved in this process are summarized in Figure 2.

## 4 Recreating the muscle niche

Numerous factors can influence the biological response of wound healing and, to date, animal models remain the predominant choice for studying the mechanisms that govern tissue homeostasis and regeneration. This preference persists because no alternative system can accurately replicate the intricate complexity of a living organism. However, the complexity of an organism also imposes a challenge for the study of specific cellular and molecular interactions. So, novel *in vitro* methods that recreate the muscle niche, such as bioengineered muscles or matrix scaffolds have been developed for studying specific biological aspects and cellular crosstalk, and as promising therapeutic platform for muscle-based diseases.

### 4.1 *In vitro* models of the muscle niche

Organoids or organ spheroids are 3D self-organized *in vitro* structures derived from stem cells that mimic the complexity of native tissues. Recent advancements in organoid research have introduced self-organizing assembloids, a novel approach with the potential to reshape our understanding of intercellular dynamics within neuromuscular organoids (Faustino Martins et al., 2020). These assembloids offer insights into the development and function of neuromuscular components by facilitating the study of complex cellular interactions. Furthermore, skeletal muscle organoids have been described with a structural organization of muscle cells replicated the sequential occurrence of multiple myogenic cell types from MuSCs to myocytes (Shin et al., 2022). This organoid also presents regenerative capacity after injury induction, emerging as an interesting *in vitro* model for studying skeletal muscle and related diseases (Shin et al., 2022). The development of mature myofibers, associated progenitors and other muscle cells can be achieved by manipulating cell fate of pluripotent stem cells (Chal et al., 2015; Chal et al., 2016). These cultures can be generated from healthy or dystrophic backgrounds and, besides offering an interesting 2D platform to study individual cell types without the influence of neighboring cells, allows engineering of different types of 3D artificial muscles (Chal et al., 2015; Chal et al., 2016). Regardless of the cell type used, self-organized organoids do not replicate the mechanical cues that recreate the architectural alignment of native skeletal muscles. To overcome this problem, pluripotent stem cells have been embedded in hydrogels and anchored between two attachment points (Gholobova et al., 2018; Maffioletti et al., 2018). This approach efficiently stimulates 3D skeletal myogenic differentiation and alignment, of both healthy and dystrophic induced pluripotent stem cells (iPSCs), into constructs that recapitulate molecular, structural, and functional muscle features. Additionally, deriving and combining isogenic ECs and PCs derived from the same iPSCs, into hydrogels generates a more physiologically relevant *in vitro* model and improves the *in vivo* survival of larger constructs (Maffioletti et al., 2018).

### 4.2 Bioengineered scaffolds and 3D bioprinting for muscle regeneration

Scaffolds and biomaterials have been exploited to mimic the natural muscle environment. In the last few decades, many advances have been made in scaffold fabrication techniques and biomaterials. Fiber orientation and suitable biomaterials are important factors for achieving the highest degree of myotube formation and tissue contractility (Guex et al., 2012), as scaffold-type has been shown to affect the behavior of MuSCs and MPs (Mahon et al., 2021) and stiffness muscle development (Levy-Mishali et al., 2008). These biomaterials can either be naturally derived such as collagen, fibrin and decellularized ECM or synthetic polymers and hybrid compounds.

3D bioprinting is another fabrication technique that has been widely studied for tissue engineering strategies and the creation of engineered muscles. This technology allows the manufacture of complex 3D constructs by combining in a bio ink: scaffold components, growth factors and even cells (Matai et al., 2020). An example of this is an implantable 3D construct composed of human primary muscle progenitor cells that successfully restored a *tibialis anterior* muscle defect and its muscle function in rat, with well-integrated vascularization and a host nerve network (Kim et al., 2018). More recently, researchers have developed highly organized 3D constructs of murine muscle progenitors. These cultures differentiate into organized muscle bundles capable of spontaneously contracting *in vitro* and restoring volumetric muscle loss damage in mice (Fornetti et al., 2023).

Recent findings demonstrated that introducing a vascular component not only models *in vivo*-like muscle architecture and physiological features but also has critical consequences on how muscle-derived fibroblasts migrate towards muscle fibers. ECs mediate indeed the induction of muscle-specific endothelium and the self-organization of muscle-derived fibroblasts into an enveloping sheath that mimics the endomysium (Bersini et al., 2018). The resulting bio-fabricated *in vitro* 3D human vascularized skeletal muscle environment also proved to be compatible with high-resolution imaging techniques, matrix deposition analyses, and cell type-specific mRNA retrieval for in-depth gene expression analyses (Bersini et al., 2018).

Another important critical condition in recreating the muscle niche is the possibility to control culture conditions. This can be achieved by organ-on-chips, i.e., platforms that usually consist of 3D arrangements of multiple integrated cell types and the presence of microfluidic channels connected to a continuous perfusion device. This mimics the native microenvironment distribution of nutrients and cellular waste removal, and overcomes the limitations associated with static 3D cultures (Leung et al., 2022). Current muscle-on-a-chip platforms can mimic cellular and tissue responses (Agrawal et al., 2017; Naik et al., 2019). These constructs induce growth of muscle cells and fibers into organized and densely-packed cylindrical muscle tissues (Agrawal et al., 2017; Naik et al., 2019) that express myogenic proteins and proteins involved in mitochondrial health and biogenesis (Naik et al., 2019). They can also recapitulate the ability to respond to cardiotoxin-induced injury through tissue structure and function changes (Agrawal et al., 2017). Then, incorporation of other muscle cells allows the development of improved constructs and a more in-depth study of cellular interactions. For instance, bioengineering vascularization of a microfluidic platform enhances muscle contraction and differentiation, while significantly upregulating angiogenic sprouting of ECs (Osaki et al., 2018).

## 5 Concluding remarks and future perspectives

In recent years, there has been a growing interest in understanding how the different muscle populations communicate with each other. This complex interplay highlights the importance of considering the muscle environment not just as a collection of individual cells, but as a dynamic ecosystem. Emerging ideas in cancer research, such as models of tumor ecology and metastasis, emphasize the importance of studying disease processes within a broader ecological context. This perspective aligns with the emerging concept of “ecological pathology”, which emphasizes the interplay between cells and their surrounding environment in disease processes (Luo, 2023) and offers potential for future exploration of how these interactions influence regeneration, particularly in diseased states.

Despite extensive research effort, the mechanisms governing these intricate cell-cell interactions remain not fully understood, also due to the recent identification of additional key players in this complex scenario like FAPs, and the observation that vessel-associated cells exert a previously unidentified role in the cellular and molecular crosstalk of the regenerating muscle niche.

Here we have provided an updated review of the functions and contributions of both MPs, FAPs and the often-overlooked vessel-associated cells. The rapid progress in technologies like single-cell RNA sequencing has indeed allowed the identification of previously undiscovered subpopulations of muscle cells and a deeper insight into those already recognized, shedding light on an even higher molecular and cellular complexity composing the muscle. Furthermore, we outlined the progress made in establishing novel experimental setups capable of mimicking this intricate environment *in vitro,* paving the way for the exploration and refinement of effective therapeutic approaches.
